# Simultaneous determination of osimertinib and almonertinib concentrations in human plasma and cerebrospinal fluid using UPLC-MS/MS method and its clinical applications

**DOI:** 10.3389/fphar.2026.1943759

**Published:** 2026-10-21

**Authors:** Jintuo Yin, Yalan Zuo, Xin Wang, Zhentao Zhang, Hongyu Yue, Jian Shi, Ying Han, Jiang Liu, Ping Liang

**Affiliations:** Department of Pharmacy, The Fourth Hospital of Hebei Medical University, Shijiazhuang, Hebei, China

**Keywords:** almonertinib, cerebrospinal fluid concentration, clinical applications, osimertinib, plasma concentration, UPLC-MS/MS

## Abstract

**Objectives:**

This study aimed to establish a sensitive analytical method to simultaneously measure concentrations of two third-generation EGFR inhibitors, osimertinib and almonertinib, in human plasma and cerebrospinal fluid. A further objective was to investigate the relationship between these drug concentrations, their penetration into the central nervous system, and clinical treatment efficacy in non-small cell lung cancer (NSCLC) patients with brain metastases.

**Methods:**

An ultra-performance liquid chromatography-tandem mass spectrometry (UPLC-MS/MS) method was developed and fully validated for the simultaneous quantification of osimertinib and almonertinib. Plasma and cerebrospinal fluid samples were collected from twenty NSCLC patients with brain metastases (7 receiving osimertinib, 13 receiving almonertinib) at steady-state trough concentration. Drug concentrations were measured, and blood-brain barrier penetration rates were calculated (cerebrospinal fluid/plasma concentration ratio × 100%). Treatment efficacy was assessed via lung CT and brain MRI using RECIST criteria.

**Results:**

The validated UPLC-MS/MS method demonstrated excellent sensitivity, specificity, and accuracy for both drugs in both matrices. Significant inter-individual variability was observed in plasma concentrations, cerebrospinal fluid concentrations, and penetration rates. For both drugs, steady-state plasma concentration showed no significant correlation with lung tumor response. However, higher cerebrospinal fluid concentration and penetration rate were positively correlated with improved treatment efficacy for brain metastases, as evidenced by better outcomes on brain MRI.

**Conclusion:**

Therapeutic drug monitoring of osimertinib and almonertinib in cerebrospinal fluid, rather than plasma alone, may provide a more relevant indicator for assessing and optimizing treatment efficacy against brain metastases in NSCLC. This study would develop personalized treatment plans for NSCLC patients with brain metastases, providing important evidence for clinical practice.

## 1 Introduction

Lung cancer is one of the malignant tumors with the highest incidence rate and mortality in the world, which seriously endangers human health. According to the Global Cancer Epidemiology Statistics (GLOBOCAN), there were approximately 2.207 million new cases of lung cancer and 1.796 million new lung cancer deaths worldwide in 2020 (Liu et al., 2021; Soin et al., 2024). Non-small Cell Lung Cancer (NSCLC) is the most common type of lung cancer worldwide, accounting for approximately 85% of all lung cancer cases. Since the approval of gefitinib in 2003, EGFR tyrosine kinase inhibitors (EGFR-TKIs) have become the first-line treatment option for EGFR mutation NSCLC patients (Ma et al., 2024; Ok et al., 2024).

Compared with early EGFR-TKIs, third-generation EGFR-TKIs not only demonstrate stronger efficacy in treating drug-resistant NSCLC, but also have advantages in reducing side effects and improving quality of life (Kadyrbayeva et al., 2024). Osimertinib, as one of the representative drugs of third-generation EGFR-TKI, has a potent inhibitory effect on common EGFR mutations such as T790M, Ex19del, and L858 R and exhibits therapeutic activity on the central nervous system (CNS) (Nishioka et al., 2024). Research had shown that osimertinib, with small relative molecular weight and good lipid solubility, could penetrate the blood-brain barrier (BBB) and control EGFR mutant NSCLC brain metastasis (Pang et al., 2024). Almonertinib was the first third-generation targeted drug independently developed in China for the treatment of EGFR mutant NSCLC, and had been approved by the China Food and Drug Administration (CFDA). As a third-generation EGFR tyrosine kinase inhibitor (EGFR-TKI), almonertinib had shown significant clinical efficacy against T790M mutation, especially in patients with locally advanced or metastatic NSCLC. Brain and spinal cord metastasis are one of the common complications of advanced NSCLC. One of the key challenges in the treatment of NSCLC patients with brain or meningeal metastases is how to effectively deliver drugs to cerebrospinal fluid and brain tissue by breaking through the BBB (Shen et al., 2021; Zhang et al., 2021a). Due to their optimized molecular structure, osimertinib and almonertinib had shown better BBB permeability as early-generation EGFR-TKI drugs, and their application in patients with brain metastases had gradually attracted attention. However, there was still limited systematic research on the permeability, drug concentration, and clinical efficacy of osimertinib and almonertinib in cerebrospinal fluid (Shen et al., 2021). In recent years, osimertinib and almonertinib had shown excellent clinical efficacy, which made them unique advantages in treating NSCLC patients with brain metastases (Zhang QC. et al., 2023). However, in clinical applications, different patients were influenced by various factors such as genotype, metabolic enzyme activity, concomitant medication use, and pathophysiological status. At present, the main methods for detecting EGFR-TKIs concentration include high-performance liquid chromatography (HPLC), high-performance liquid chromatography tandem mass spectrometry (HPLC-MS/MS), and ultra-high-performance liquid chromatography tandem mass spectrometry (UPLC-MS/MS). These methods are not only widely used in monitoring drug concentrations in plasma, but also gradually applied in the study of drug concentrations in cerebrospinal fluid, especially in evaluating the ability of EGFR-TKI drugs to penetrate the BBB (Zhao et al., 2018). UPLC-MS/MS method, with excellent sensitivity, specificity, and rapid analytical ability, could accurately detect the concentrations of osimertinib and almonertinib in plasma and cerebrospinal fluid at extremely low concentrations. It was particularly suitable for simultaneous determination of drug concentrations in these two biological samples, providing a basis for clinical treatment (Zhao et al., 2018; Hsu et al., 2025).

In the study, the concentrations of osimertinib and almonertinib in human plasma and cerebrospinal fluid were systematically measured for the first time by optimizing UPLC-MS/MS method, aiming to evaluate their distribution in plasma and the central nervous system and provide monitoring data (Guan et al., 2019). Secondly, imaging results were collected, including lung CT and brain MRI results, to evaluate the correlation between blood drug concentration, cerebrospinal fluid concentration or permeability, and efficacy, which could develop personalized treatment plans for NSCLC patients with brain metastases, and provide an important basis for clinical practice.

## 2 Materials and methods

### 2.1 Chemicals and reagents

The standards of osimertinib, almonertinib, and glimepiride were provided by the China National Institute for Food and Drug Control (Beijing, China). All of their purities were greater than 99.8%. Methanol and acetonitrile (HPLC grade) were purchased from Aladdin Reagent (Shanghai) Co., Ltd. (Shanghai, China). Formic acid (analytical grade) was also bought from Aladdin Reagent (Shanghai) Co., Ltd. (Shanghai, China). Purified water was from Hangzhou Wahaha Group Co., Ltd. (Hangzhou, China).

### 2.2 LC-MS conditions

Samples were analyzed on a HPLC system (Agilent Technologies, CA, United States of America) equipped with an autosampler, a quaternary solvent delivery system, and a column temperature chamber. HPLC separations were carried out on a BEH C18 chromatographic column (50 mm × 2.1 mm, 1.7 μm, Waters Corporation, United States of America) at 40 °C. Gradient elution was performed using water containing 0.1% (v/v) formic acid (A) and acetonitrile (B) as mobile phases. The flow rate was processed at 0.3 mL/min as follows: 0–0.01 min, 20% B; 0.01–1.5 min, 80% A; 1.5–4 min, 80% B; 4–4.1 min, 20% B; and 4.1–5 min, 20%. The injected volume for all samples was 10 μL.

For detection and quantification, MS detection was performed using an AB Sciex Triple Quad 5,500 (AB SCIEX, United States of America). The ion spray voltages were set to 5500 V, the source temperature was maintained at 500 °C, the ion source gas (gas 1) pressure was 55 psi, the ion source gas (gas 2) pressure was 55 psi, the collision cell-exit potentials were 15 V, and the entrance potentials were 11 V. The MS instrument was operated in MRM mode and monitored in positive ion mode. The declustering potential (DP) and collision energy (CE) of the quantitative-optimization mode for the drugs in the study were described in Table 1.

**TABLE 1:** The DP and CE of the quantitative-optimization mode for the drugs.

Name	*m/z*	DP	CE
Osimertinib	500.2→72.1	163 V	65 V
Almonertinib	506.1→72.2	140 V	80 V
Glimepiride	491.2→352.0	60 V	20 V

### 2.3 Preparation of analytes and internal standard solutions

#### 2.3.1 Preparation of osimertinib and almonertinib stock solutions

Standard stock solutions of osimertinib and almonertinib were prepared in 50% methanol-water at approximate concentrations of 1.02 mg/mL. For each analyte, two separate weighings (osimertinib: 10.18 and 10.23 mg; almonertinib: 10.27 and 10.13 mg) were diluted to 10 mL to yield paired stock solutions, designated for calibration standards and quality control (QC) samples, respectively.

#### 2.3.2 Preparation of osimertinib and almonertinib working solutions

Calibration and quality control samples for osimertinib/almonertinib in plasma and cerebrospinal fluid were prepared by diluting stock solutions with 50% aqueous methanol. For plasma, the calibration curve ranged from 1 to 1,000 ng/mL (1, 3, 10, 30, 100, 300, 1,000 ng/mL), with quality controls at 1, 3, 100, and 800 ng/mL. For cerebrospinal fluid, the calibration curve ranged from 1 to 100 ng/mL (1, 3, 5, 10, 30, 50, 100 ng/mL), with quality controls at 1, 3, 10, and 80 ng/mL.

#### 2.3.3 Preparation of internal standard stock solution and working solution

Stock and working solutions of the internal standard glimepiride were prepared by accurately weighing 10.16 mg into a 10 mL volumetric flask (Yang, 2023), dissolving it with 50% methanol-water, and diluting to volume to obtain a 1.016 mg/mL stock solution. A 100 µL aliquot of this stock was then precisely diluted with 50% methanol-water to prepare a 100 ng/mL internal standard working solution.

All samples were stored in a −20 °C refrigerator for until analysis.

### 2.4 Processing of standard curves and QC samples for plasma and cerebrospinal fluid

Aliquots (380 µL) of plasma or cerebrospinal fluid were spiked with 20 µL of the corresponding osimertinib/almonertinib standard or QC solution. A 20 µL aliquot of each spiked matrix was then mixed with 20 µL of the internal standard glimepiride solution (100 ng/mL) and 100 µL of methanol. After vortexing and centrifuging (11,000 rpm, 5 min), 30 µL of the supernatant was diluted with 270 µL of 50% methanol-water. Following vortex mixing, 1 µL was injected for LC analysis (Xi et al., 2020).

### 2.5 Method validation

#### 2.5.1 Specificity and selectivity

By comparing the chromatograms of blank plasma, blank plasma plus osimertinib/almonertinib, and internal standard glimepiride, as well as the chromatograms of blank cerebrospinal fluid, blank cerebrospinal fluid plus osimertinib/almonertinib, and internal standard glimepiride, the interference level of blank matrix on analytes and internal standards could be evaluated.

Six samples were tested in parallel, and the processing methods were as described in “2.4 Processing of plasma and cerebrospinal fluid standard curves and quality control samples.”

#### 2.5.2 Standard curve and linear range

Calibration standards were prepared by spiking 380 µL of blank plasma or cerebrospinal fluid with 20 µL of the corresponding osimertinib/almonertinib calibration working solution. The final concentration ranges were 1–1,000 ng/mL in plasma and 1–100 ng/mL in cerebrospinal fluid. Following the sample processing method described in Section 2.4, the peak area ratios (Y) *versus* concentrations (X) were analyzed by weighted (1/X^2^) least squares regression to establish the calibration curve.

#### 2.5.3 Accuracy and precision

QC samples were prepared by spiking 380 µL of blank plasma or cerebrospinal fluid with 20 µL of the corresponding osimertinib/almonertinib QC working solution. Final concentrations were as follows: in plasma—the lowest limit of quantification (LLOQ) (1 ng/mL), Low (3 ng/mL), Medium (100 ng/mL), High (800 ng/mL); in cerebrospinal fluid—LLOQ (1 ng/mL), Low (3 ng/mL), Medium (10 ng/mL), High (80 ng/mL). Six replicates at each level were prepared and processed according to the method described in Section 2.4.

#### 2.5.4 Matrix effect

Matrix effect was evaluated by comparing the peak area ratios of osimertinib/almonertinib and the internal standard (glimepiride) in biological matrices *versus* those in water (neat solution). For both plasma and cerebrospinal fluid, six replicates of low and high QC samples (plasma: 3 and 800 ng/mL; cerebrospinal fluid: 3 and 80 ng/mL) were prepared from blank matrix and processed as described in Section 2.4. The corresponding peak areas from samples prepared in water were also recorded. The matrix factor (MF) for each analyte and the IS was calculated as the ratio of peak area ratios (with matrix/without matrix). The normalized MF (MFanalyte/MFIS) was determined, and the matrix effect was considered acceptable if the deviation did not exceed 15%.

#### 2.5.5 Extraction recovery rate

Three concentration control samples (3, 100, and 800 ng/mL) of plasma and three concentration control samples (3, 10, and 80 ng/mL) of cerebrospinal fluid in osimertinib/almonertinib were prepared according to the processing method in Section 2.4, with six samples for each control concentration. At the same time, blank plasma or cerebrospinal fluid was taken and treated with protein precipitation. The test substance control solution and internal standard solution was then added to the supernatant. Six replicates were prepared in parallel for each concentration and analyzed by injection. The recovery rate was calculated based on the peak area ratio of the two treatment methods for each concentration, and the RSD for each concentration should be less than 15%.

#### 2.5.6 Stability

Low and high concentration (3, 800 ng/mL) quality control samples of plasma and low and high concentration (3, 80 ng/mL) quality control samples of cerebrospinal fluid in osimertinib/almonertinib were prepared according to the sample processing method in “2.4,” with six samples for each quality control concentration.

Room temperature stability: All samples were left at room temperature for 6 h; Long-term stability: All samples were stored at −80 °C for 30 days; Repeated freeze-thaw stability: All samples were subjected to four repeated freeze-thaw cycles at −80 °C and room temperature.

According to the standard curve of the day, the concentrations of the quality control samples were analyzed, and the measured concentrations should be less than ±15% of the labeled concentration RE, with RSD less than 15%.

### 2.6 Exposure-response relationship of osimertinib and almonertinib

#### 2.6.1 Research object

The study included twenty patients with EGFR mutant NSCLC brain metastases (7 treated with osimertinib and 13 treated with almonertinib). The research plan had been approved by the Ethics Committee of the Fourth Hospital of Hebei Medical University (Ethics Approval Number: 2022KS008). The strict implementation of ethical approval ensured the compliance of research and the patient’s right to know, and relevant ethical and legal norms were followed during the research process. Supplementary data about the patients was provided in Supplementary materials.

#### 2.6.2 Sample collection

The collection of samples included plasma and cerebrospinal fluid samples in osimertinib/almonertinib, both of which were taken when the patient reached drug steady state. To ensure the accuracy of drug concentration determination, all samples were collected uniformly in the morning before the patient’s daily administration to obtain steady-state trough concentration (Cmin). Plasma and cerebrospinal fluid samples were collected at the same time to ensure synchronous analysis of drug concentrations in both samples.

#### 2.6.3 Sample processing and measurement

Patient samples were processed and analyzed as specified in Section 2.2. The concentration of osimertinib/almonertinib in each sample was determined by applying the measured peak area ratio (Y) to the established calibration curve. The LLOQ was 1 ng/mL for both plasma and cerebrospinal fluid.

To assess CNS penetration, the BBB penetration rate was calculated using the following formula:

Penetration Rate (%) = (Cerebrospinal Fluid Concentration/Plasma Concentration) × 100%

This ratio was a critical pharmacokinetic parameter for evaluating drug distribution into the CNS, particularly in the treatment of NSCLC brain metastases.

#### 2.6.4 Collection of basic patient information

Basic clinical data of EGFR mutant NSCLC patients with brain metastases who met the research criteria was collected, including age, gender, weight, BMI, medical history, clinical manifestations, primary cancer histology, treatment status, comprehensive evaluation results of patients at the time of starting to use osimertinib/almonertinib and follow-up, including disease changes, imaging results (lung CT, brain MRI), cerebrospinal fluid test results, EGFR gene mutation status changes, treatment methods, and their start and end dates.

#### 2.6.5 Collection and evaluation of imaging data

1. Data Collection: Baseline and follow-up imaging studies were performed for each patient before and after a defined course of treatment with osimertinib or almonertinib.

Chest CT: Scans were conducted to assess changes in the size of the primary lung tumor and the status of metastatic lesions.

Brain MRI: Scans were performed to evaluate changes in brain and meningeal metastases, with particular attention to lesion characteristics and implications for cerebrospinal fluid barrier permeability.

2. Response Evaluation: Treatment efficacy was assessed by comparing pre- and post-therapy images to quantify volumetric changes in both lung and brain lesions. Tumor response was classified according to the Response Evaluation Criteria in Solid Tumors (RECIST) as complete response (CR), partial response (PR), stable disease (SD), or progressive disease (PD). Clinical efficacy was defined as the achievement of CR, PR, or SD. This imaging-based evaluation provided a direct assessment of drug efficacy, especially in patients with brain metastases.

#### 2.6.6 Statistical methods

SPSS software was used for data statistical analysis, and the measurement data was expressed as mean ± standard deviation (x ± s). Graphics were drawn using GraphPad Prism 9.5.0 software. The comparison between the two groups was analyzed using a t-test. Multiple group comparisons were conducted using one-way ANOVA analysis of variance. P < 0.05 indicated a statistically significant difference.

## 3 Results

### 3.1 Method validation

#### 3.1.1 Selectivity

The UPLC-MS/MS analysis yielded chromatograms for osimertinib and almonertinib in plasma and cerebrospinal fluid (Figure 1). Retention times were consistent across matrices: in plasma, almonertinib, the internal standard (glimepiride), and osimertinib eluted at 1.351, 2.198, and 1.512 min, respectively; corresponding times in cerebrospinal fluid were 1.350, 2.197, and 1.517 min. The method demonstrated good selectivity, as the response from interfering components at the analyte retention times was <20% of the LLOQ response and <5% of the internal standard response, confirming no significant interference from endogenous matrix components.

**FIGURE 1:**
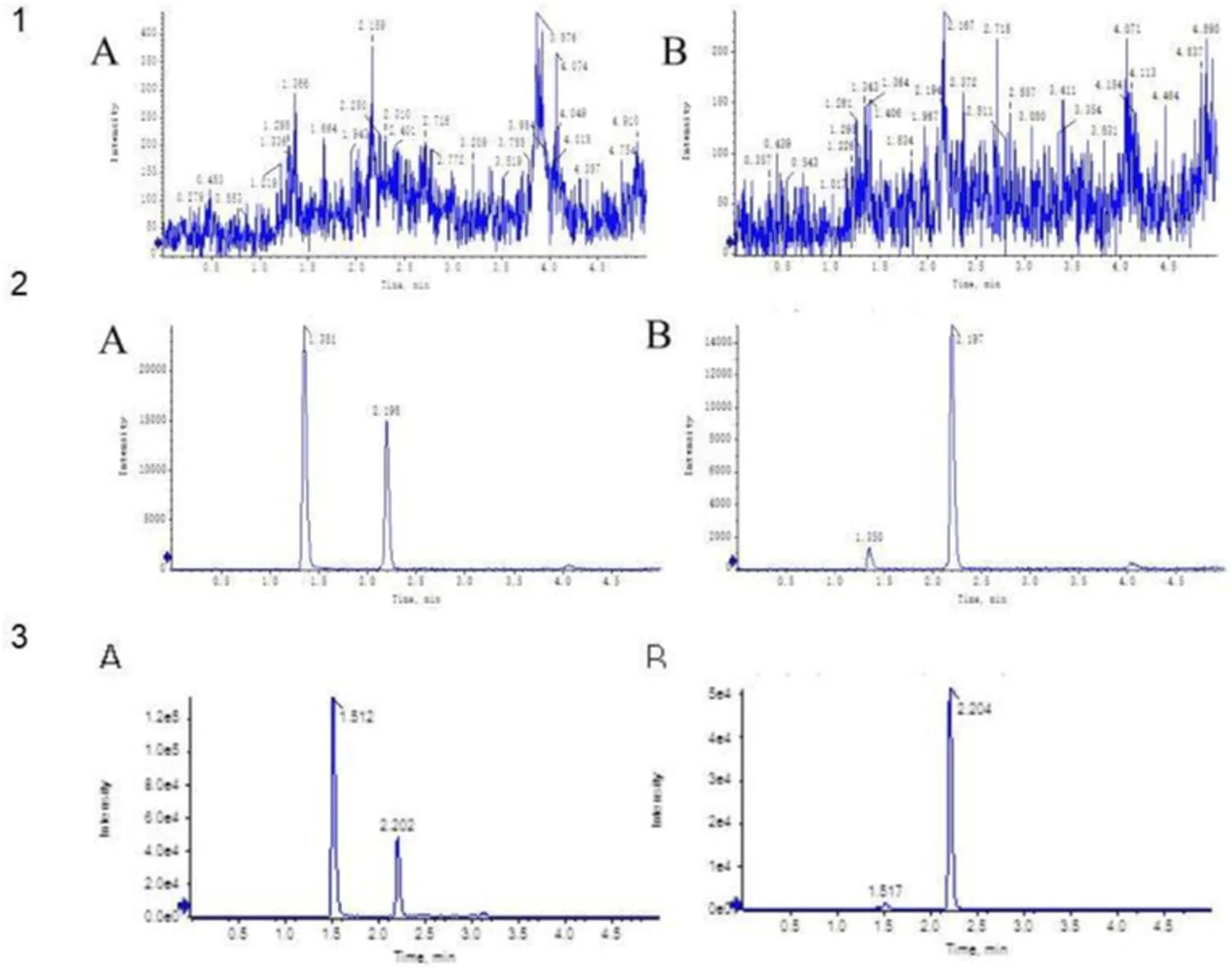
Chromatogram of plasma [1**(A)**-blank, 2**(A)**-osimertinib and 3**(A)**-almonertinib] and cerebrospinal fluid [1**(B)**- blank plasma, 2**(B)**-osimertinib, 3**(B)**-almonertinib].

#### 3.1.2 Standard curve

The results showed that there was a good linear relationship between osimertinib in plasma and cerebrospinal fluid, with correlation coefficients of 0.995 and 0.997 (see Table 2), while almonertinib had a good linear relationship in plasma and cerebrospinal fluid, with correlation coefficients of 0.994 and 0.998, respectively (see Table 2).

**TABLE 2:** Calibration range, typical calibration curve linear regression equation, and correlation coefficient (R^2^)of osimertinib/almonertinib.

Drug	Sample	Calibration range (ng/mL)	Standard curve equation	Correlation coefficient (R^2^)
Osimertinib	Plasma	1–1,000	Y = 0.0069X + 0.0159	0.995
	Cerebrospinal fluid	1–100	Y = 0.0098X + 0.0224	0.997
Almonertinib	Plasma	1–1,000	Y = 0.0026X + 0.0185	0.994
	Cerebrospinal fluid	1–100	Y = 0.0075X + 0.0657	0.998

#### 3.1.3 Precision and accuracy

The LLOQ for osimertinib and almonertinib was established at 1 ng/mL in both plasma and cerebrospinal fluid. The intra- and inter-batch precision (RSD) for quality control samples at low, medium, and high concentrations in plasma (3, 100, 800 ng/mL) and cerebrospinal fluid (3, 10, 80 ng/mL) was ≤15%, while accuracy (RE) was within ±10%, meeting the validation criteria of the Chinese Pharmacopoeia (Table 3).

**TABLE 3:** Intra- and inter-day precision and accuracy of plasma and cerebrospinal fluid concentration of osimertinib/almonertinib (Mean ± SD, n = 6).

Drug	Sample	Theoretical concentration (ng/mL)	Intra-day precision			Inter-day precision		
			Measured concentration (ng/mL)	Precision RSD (%)	Accuracy RE (%)	Measured concentration (ng/mL)	Precision RSD (%)	Accuracy RE (%)
Osimertinib	Plasma	1	1.09 ± 0.04	7.72	9.32	1.08 ± 0.08	2.61	8.02
		3	2.85 ± 0.19	7.73	−5.13	2.95 ± 0.22	7.13	−1.73
		100	96.72 ± 5.29	5.01	−3.34	95.57 ± 4.67	3.87	−4.43
		800	859.58 ± 8.41	0.86	7.43	852.50 ± 8.63	1.76	6.56
	Cerebrospinal fluid	1	1.06 ± 0.07	10.67	6.38	1.05 ± 0.13	2.05	4.67
		3	3.15 ± 0.22	7.33	5.31	3.12 ± 0.23	5.97	4.32
		10	9.72 ± 0.71	5.93	−2.83	9.69 ± 0.16	1.47	−3.16
		80	81.08 ± 5.04	5.08	1.44	82.15 ± 2.62	2.38	2.68
Almonertinib	Plasma	1	0.98 ± 0.09	12.09	−1.73	1.02 ± 0.11	7.59	4.65
		3	3.16 ± 0.38	9.41	5.46	3.24 ± 0.24	3.73	8.21
		100	109.15 ± 7.21	6.34	9.12	103.91 ± 6.41	7.91	3.94
		800	811.68 ± 21.04	2.79	1.59	813.85 ± 18.22	1.61	1.73
	Cerebrospinal fluid	1	1.04 ± 0.09	9.92	4.66	1.03 ± 0.12	2.38	3.12
		3	3.13 ± 0.39	10.82	2.93	3.09 ± 0.25	2.93	3.31
		10	10.77 ± 1.13	9.53	7.73	10.93 ± 1.84	2.81	9.33
		80	81.51 ± 2.69	4.06	1.95	82.18 ± 3.36	5.82	2.73

#### 3.1.4 Matrix effect

The matrix effects of low and high concentrations (3 ng/mL and 800 ng/mL for plasma and 3 ng/mL and 80 ng/mL for cerebrospinal fluid) of osimertinib and almonertinib were calculated from six blank plasma and cerebrospinal fluid samples, and the matrix effects of the QC samples were between 86.98% and 104.23%, which met the guidelines for the validation of quantitative analysis methods for biological samples. This indicated that under this analytical method, matrix effects did not affect the accuracy of the measurement results (see Table 4).

**TABLE 4:** Matrix effect of plasma and cerebrospinal fluid concentration of osimertinib/almonertinib (Mean ± SD, n = 6).

Drug	Sample	Theoretical concentration (ng/mL)	Matrix effect (%)	RSD (%)
Osimertinib	Plasma	3	100.78 ± 3.75	3.84
		800	99.67 ± 3.89	3.92
	Cerebrospinal fluid	3	104.23 ± 4.21	4.05
		80	97.91 ± 3.25	3.38
Almonertinib	Plasma	3	86.89 ± 3.28	3.86
		800	103.45 ± 3.89	5.71
	Cerebrospinal fluid	3	98.64 ± 4.63	4.76
		80	103.61 ± 5.23	5.14

#### 3.1.5 Extraction recovery rate

The extraction recoveries for osimertinib and almonertinib from plasma and cerebrospinal fluid are summarized in Table 5. For plasma QC samples at low, medium, and high concentrations, recoveries ranged from 101.15% to 106.32%. The mean recoveries were 104.1% in plasma and 102.9% in cerebrospinal fluid. The high and consistent recovery rates across both matrices demonstrated the reliability of the sample pretreatment and analytical method.

**TABLE 5:** Extraction recovery of plasma and cerebrospinal fluid concentration of osimertinib/almonertinib (Mean ± SD, n = 6).

Drug	Sample	Theoretical concentration (ng/mL)	Extraction recovery rate (%)	RSD (%)
Osimertinib	Plasma	3	106.32 ± 10.64	10.08
		100	103.41 ± 3.39	3.36
		800	102.35 ± 3.29	3.24
	Cerebrospinal fluid	3	103.41 ± 9.51	9.22
		10	98.95 ± 2.41	2.45
		80	101.82 ± 5.27	5.25
Almonertinib	Plasma	3	107.73 ± 7.96	7.46
		100	103.56 ± 3.39	6.43
		800	102.31 ± 5.43	5.38
	Cerebrospinal fluid	3	100.58 ± 9.59	9.54
		10	103.89 ± 8.68	8.49
		80	97.62 ± 3.23	3.37

#### 3.1.6 Stability

The stability of osimertinib and almonertinib in plasma and cerebrospinal fluid was evaluated under various conditions, with results summarized in Table 6. For both low and high concentration samples (plasma: 3 and 800 ng/mL; cerebrospinal fluid: 3 and 80 ng/mL), the precision RSD was ≤15% and the relative deviation (RE) was within ±15% of the nominal concentration. These data demonstrated that the analytes remained stable in both matrices under short-term (6 h at 25 °C), long-term (30 days at −80 °C), and through multiple freeze-thaw cycles (4 cycles at −80 °C).

**TABLE 6:** Stability of plasma and cerebrospinal fluid concentration of osimertinib/almonertinib (Mean ± SD, n = 6).

Drug	Sample	Storage conditions	Theoretical concentration (ng/mL)	Measured concentration (ng/mL)	RSD (%)	RE (%)
Osimertinib	Plasma	25 °C, 6 h	3	3.01	7.46	0.59
			800	857.21	3.46	7.15
		−80 °C, 30 days	3	3.25	8.43	4.73
			800	820.47	3.72	2.63
		Freeze-thaw cycle 4 times	3	3.12	4.09	3.48
			800	802.69	5.78	0.39
Osimertinib	Cerebrospinal fluid	25 °C, 6 h	3	3.11	6.73	3.57
			80	85.24	3.31	6.68
		−80 °C, 30 days	3	3.27	7.03	9.02
			80	82.13	2.55	2.73
		Freeze-thaw cycle 4 times	3	3.23	2.54	7.91
			80	77.98	3.31	−2.52
Almonertinib	Plasma	25 °C, 6 h	3	3.23	10.53	7.82
			800	787.54	3.93	−1.68
		−80 °C, 30 days	3	3.06	9.15	1.97
			800	758.81	7.27	−5.12
		Freeze-thaw cycle 4 times	3	2.86	9.92	−4.65
			800	764.28	6.31	−4.52
Almonertinib	Cerebrospinal fluid	25 °C, 6 h	3	3.18	8.32	6.25
			80	78.87	3.73	−1.42
		−80 °C, 30 days	3	3.19	6.15	6.43
			80	76.32	6.91	−4.69
		Freeze-thaw cycle 4 times	3	3.13	8.53	3.8
			80	82.94	3.55	3.78

### 3.2 Clinical case analysis

#### 3.2.1 Observation of curative effect

General characteristics of patients were in Supplementary materials, and clinical characteristics of patients were shown in Stable 1 and Stable 2.

Representative imaging findings from patients treated with osimertinib or almonertinib are summarized as follows and illustrated in Figure 2.

**FIGURE 2:**
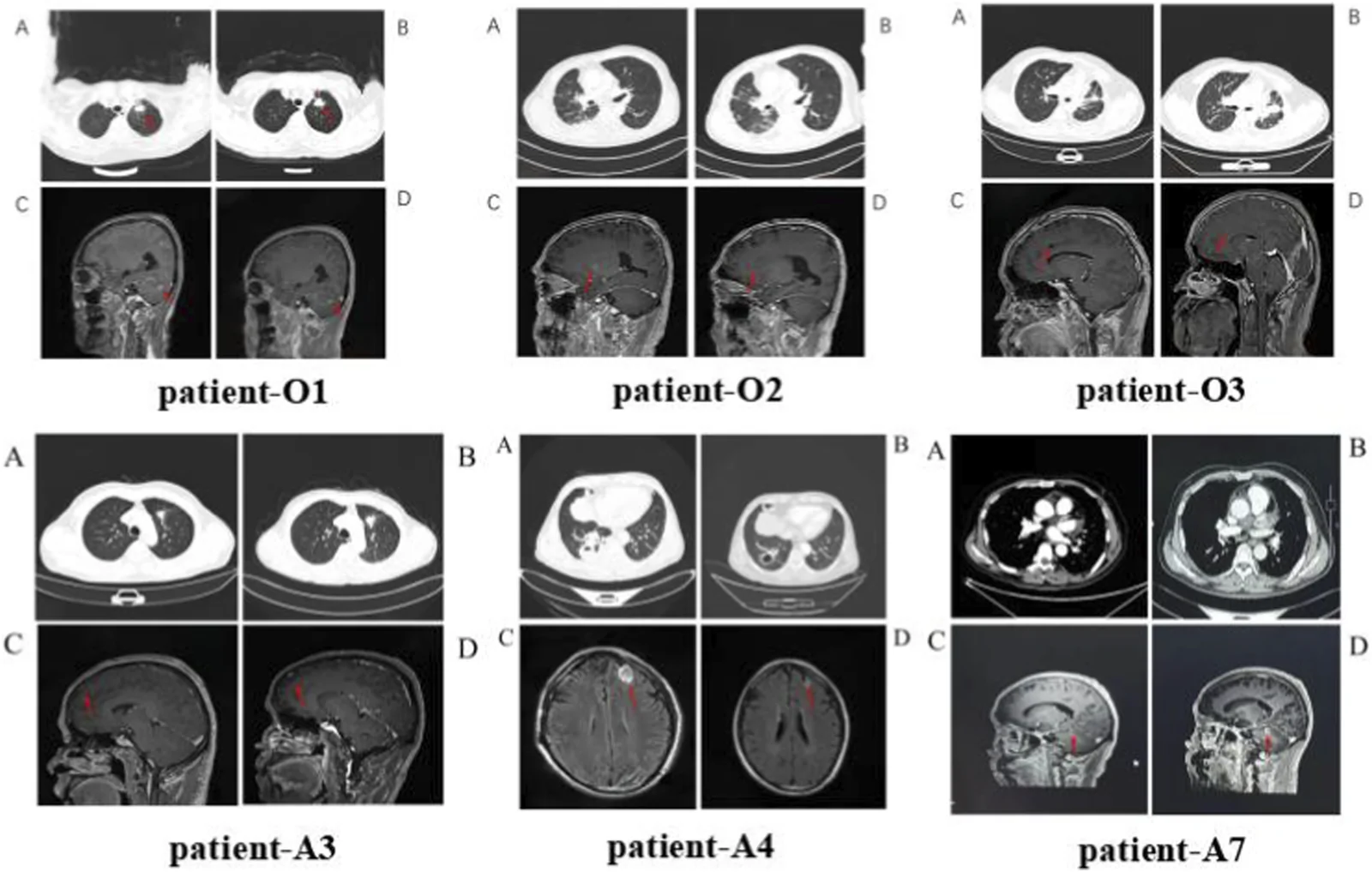
Lung CT and brain MRI of patient-O1, O2, O3 and patient-A3, A4, A7 **(A)** Lung CT scan before medication; **(B)** Lung CT scan after medication; **(C)** Brain MRI before medication; **(D)** Brain MRI after medication.

Osimertinib-treated patients (n = 7): Among the seven patients, a variable response was observed. Patients O1, O4, and O7 demonstrated stable disease (SD) in both lung and brain on follow-up imaging. Patients O2 and O3 achieved partial response (PR) in brain metastases while maintaining stable lung disease. Notably, patients O5 and O6 showed a partial response (PR) in lung lesions with stable intracranial disease, highlighting the heterogeneity of treatment response. Overall, osimertinib provided effective disease control in the majority of patients.

Almonertinib-treated patients (n = 13): A more diverse response pattern was observed. Patients A1, A6, A8, A9, A11, and A12 achieved stable disease (SD) in both lung and brain. Patients A4, A5, and A7 had a partial response (PR) in brain lesions; among these, A4 and A7 also had stable lung disease, while A5 demonstrated SD in the lung. Patient A10 showed a PR in lung lesions but experienced PD in the brain, underscoring the potential disconnect between systemic and intracranial efficacy. Patients A2, A3, and A13 experienced progressive disease (PD), with A13 specifically showing meningeal metastasis progression.

#### 3.2.2 Plasma concentration and cerebrospinal fluid concentration in patients

The CNS penetration of osimertinib and almonertinib was evaluated in NSCLC patients with brain metastases by measuring drug concentrations in matched plasma and cerebrospinal fluid samples using UPLC-MS/MS, shown in Table 7.

**TABLE 7:** Plasma and cerebrospinal fluid concentration measurements of osimertinib/almonertinib and assessment of patient efficacy.

Drug	Patient	Plasma concentration (ng/mL)	Cerebrospinal fluid concentration (ng/mL)	Permeability rates (%)	Lung CT	Brain MRI	Lung lesion changes	Brain lesion changes
Osimertinib	O1	182.0	2.2	1.21	SD	SD	↓7%	↓9%
	O2	240.4	7.8	3.24	SD	PR	↓10%	↓ 33%
	O3	395.9	24.9	6.29	SD	PR	↓15%	↓32%
	O4	245	3.11	1.27	SD	SD	↓17%	0%
	O5	67.9	2.72	4.01	PR	SD	↓60%	0%
	O6	45.3	1.54	3.4	PR	SD	↓50%	0%
	O7	78.9	2.27	2.9	SD	SD	0%	↓20%
Almonertinib	A1	225.11	2.08	0.92	SD	SD	↓17%	↓16%
	A2	128.94	4.51	3.49	PD	PD	↑23%	↑26%
	A3	186.77	0.64	0.34	PD	PD	↑22%	↑25%
	A4	268.21	30.04	11.20	PR	PR	↓37%	↓33%
	A5	100.86	31.13	30.86	SD	PR	↓29%	↓33%
	A6	343.23	1.54	0.45	SD	SD	↓8%	↓9%
	A7	200.34	29.83	14.89	SD	PR	↓6%	↓34%
	A8	82.4	1.86	2.26	SD	SD	↓9%	↑7%
	A9	136.7	3.09	2.26	SD	SD	0%	↓2%
	A10	249	1.1	0.44	PR	PD	↓29%	↑38%
	A11	173	2.13	1.23	SD	SD	0%	↓19%
	A12	144	1.64	1.14	SD	SD	↓7%	0%
	A13	120	0.71	0.59	SD	PD	↓9%	Meningeal metastasis

In seven patients treated with osimertinib, the mean plasma concentration was 179.46 ng/mL (range: 45.30–395.87 ng/mL) and the mean cerebrospinal fluid concentration was 6.35 ng/mL (range: 1.54–24.88 ng/mL). The individual CNS penetration ratios (CSF/Plasma × 100%) ranged from 1.21% to 6.29%, with a mean of 3.23%.

Similarly, in thirteen patients receiving almonertinib, the mean plasma concentration was 182.19 ng/mL (range: 82.40–343.23 ng/mL) and the mean cerebrospinal fluid concentration was 8.39 ng/mL (range: 0.64–31.13 ng/mL). The penetration ratios varied from 0.34% to 30.86%, yielding a mean of 5.92%.

#### 3.2.3 Correlation analysis between patient-measured concentration and therapeutic efficacy

As shown in Figure 3, the relationship between drug exposure and treatment response was assessed separately for osimertinib (Figures 3A–C) and almonertinib (Figures 3D–F), with response groups (PR, SD, PD) classified according to intracranial tumor response on brain MRI using RECIST criteria.

**FIGURE 3:**
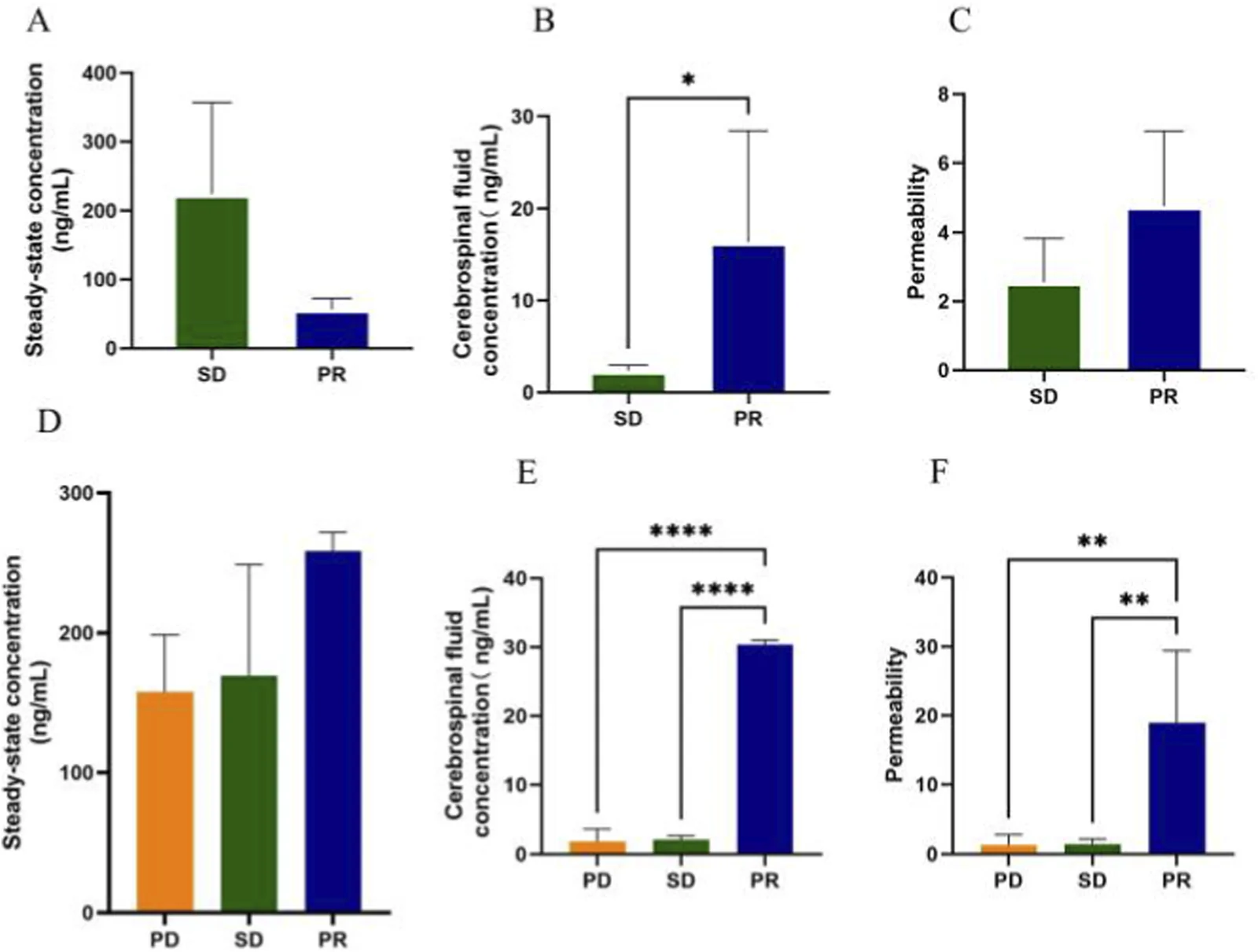
Plasma concentration, cerebrospinal fluid concentration and blood-brain barrier permeability of osimertinib/almonertinib in different therapeutic groups **(A)** -plasma of osimertinib, **(B)** -cerebrospinal fluid of osimertinib, **(C)** -blood-brain barrier permeability of osimertinib, **(D)** -plasma of almonertinib, **(E)** −cerebrospinal fluid of almonertinib, **(F)** - blood-brain barrier permeability of almonertinib) Response groups (PR, SD, PD) are classified based on intracranial tumour response assessed by brain MRI according to RECIST criteria. (*P < 0.05, **P < 0.01, ****P < 0.001)

For osimertinib (n = 7), steady-state trough plasma concentrations (Figure 3A) showed no significant association with intracranial response (P > 0.05), although patients with PR in brain lesions (O2, O3) tended to have numerically higher CSF concentrations and penetration ratios. However, due to the small sample size, no statistically significant differences were detected among the response groups for CSF concentration (Figure 3B) or penetration rate (Figure 3C).

For almonertinib (n = 13), lung CT responses (PR, n = 2; SD, n = 8; PD, n = 3) were not correlated with plasma trough concentrations (Figure 3D, P > 0.05). In contrast, for brain MRI responses (PR, n = 3; SD, n = 7; PD, n = 3), patients achieving PR had significantly higher cerebrospinal fluid concentrations (Figure 3E, P < 0.001) and penetration ratios (Figure 3F, P < 0.01) compared to those with SD or PD. No significant difference in CSF concentration or penetration ratio was observed between the SD and PD groups (both P > 0.05). Notably, patient A10, who achieved PR in lung lesions but experienced PD in the brain, exhibited an extremely low CSF concentration (1.1 ng/mL) and penetration rate (0.44%), further underscoring that adequate CNS drug exposure is critical for intracranial efficacy, whereas plasma concentration alone is a poor predictor of brain metastasis control.

These findings, derived directly from the comparative analyses in Figure 3, highlight that CSF drug concentration and blood-brain barrier penetration, rather than plasma trough levels, are the key pharmacokinetic determinants for intracranial tumor response in patients with EGFR-mutant NSCLC and brain metastases.

## 4 Discussion

### 4.1 Method establishment and validation

A selective and sensitive UPLC-MS/MS method was successfully developed and validated for the quantification of osimertinib and almonertinib in human plasma and cerebrospinal fluid. Previous studies had reported individual bioanalytical methods for quantifying osimertinib or other EGFR-TKIs in plasma using LC-MS/MS or UPLC-MS/MS. Several methods had also been developed for measuring osimertinib in cerebrospinal fluid, and a separate method had been reported for almonertinib in plasma (Irie et al., 2019; Leeuw et al., 2023; Xu et al., 2024; Li et al., 2023; Li et al., 2022a). However, to the best of our knowledge, this is the first study to simultaneously quantify both osimertinib and almonertinib in paired human plasma and CSF samples using a single validated method, enabling a direct head-to-head comparison of their central nervous system penetration characteristics**.** In blank plasma and blank cerebrospinal fluid, no significant endogenous interference was observed at the retention time of the peaks of osimertinib, almonertinib and the internal standard glimepiride, which ensured the specificity of the quantification of the analyte. Our specificity results were consistent with those reported in previous method validation studies, where no significant interference from endogenous matrix components was observed (Xu et al., 2024; Li et al., 2023). The linear relationship of the standard curve was good in the clinically relevant concentration range of plasma (1–1,000 ng/mL) and cerebrospinal fluid (1–100 ng/mL), and all coefficients of determination R^2^ were greater than 0.994, which could cover the actual steady-state plasma concentrations in the enrolled patients. The lower limit of quantification (LLOQ) of 1 ng/mL for both drugs in both matrices was comparable to or better than previously reported methods for osimertinib and almonertinib (Irie et al., 2019; Leeuw et al., 2023; Li et al., 2022a). The intra-assay and inter-assay precision (relative standard deviation ≤15%) and relative error (relative error controlled within ±10%) at the lower limit of quantification, low, medium and high-quality control concentrations met the relevant guidelines for bioanalysis, demonstrating that the repeatability of the test method was reliable. These precision and accuracy results were well within the acceptance criteria of the FDA and EMA bioanalytical guidelines, and are comparable to those reported in similar studies (Xu et al., 2024; Li et al., 2023). The matrix effect of the two analytes ranged from 86.89% to 104.23%, and the relative standard deviations were lower than 10%, which met the acceptable range, indicating that the ion suppression or ion enhancement effect caused by endogenous components in plasma and cerebrospinal fluid was negligible. This finding is consistent with previous reports where matrix effects for EGFR-TKIs in plasma and CSF were within the acceptable range of ±15% (Leeuw et al., 2023; Li et al., 2023). The average extraction recoveries of the two tyrosine kinase inhibitors were about 103%, indicating that the protein precipitation pretreatment method was stable and efficient, and there was no significant loss of the analyte. In addition, osimertinib and almonertinib have good stability under short-term storage at room temperature, long-term cryopreservation at −80 °C and repeated freezing and thawing conditions, facilitating flexible transport and batch testing of clinical samples. Similar stability profiles had been reported previously for both drugs under comparable storage conditions (Irie et al., 2019; Li et al., 2022a). This method can simultaneously determine osimertinib and almonertinib in two biological matrices, greatly improving the detection efficiency and facilitating the direct comparison of central nervous system penetration characteristics between the two drugs in clinical practice. Validation confirmed acceptable accuracy, precision, matrix effect, recovery, and stability, meeting bioanalytical guidelines. More importantly, we demonstrate that CSF drug concentrations, rather than plasma concentrations, are strongly associated with intracranial tumor control, providing novel evidence for therapeutic drug monitoring (TDM) of EGFR mutant NSCLC complicated by brain metastases. This exposure-efficacy relationship has not been previously reported in studies that focused primarily on method development (Irie et al., 2019; Li et al., 2022a), highlighting the unique clinical contribution of our work beyond the analytical method itself.

### 4.2 Plasma/cerebrospinal fluid concentration and penetration ratio

In this study, we systematically compared systemic and intracranial drug exposure levels between osimertinib and almonertinib by collecting clinically matched paired plasma-cerebrospinal fluid samples at steady state. The mean trough plasma concentrations were 179.46 ng/mL for osimertinib and 182.19 ng/mL for almonertinib, which were similar and consistent with therapeutic drug monitoring data in patients with previous non-metastatic non-small cell lung cancer, confirming that the extent of systemic drug accumulation was similar after conventional oral administration of the two drugs. However, there were significant differences in CSF drug exposure and blood-brain barrier penetration efficiency between the two groups. The mean cerebrospinal fluid concentration and blood-brain barrier penetration rate of almonertinib were 8.39 ng/mL and 5.92%, respectively, which were significantly higher than those of osimertinib (6.35 ng/mL and 3.23%). Our observed osimertinib penetration rate of 3.23% was comparable to the 2.5% penetration rate previously reported by Nanjo and colleagues (Nanjo et al., 2018) in patients with leptomeningeal metastases, and was also consistent with findings from other recent studies (Irie et al., 2019; Leeuw et al., 2023). This difference stems from the physicochemical properties and transporter properties of the two drugs themselves: almonertinib has a lower affinity for P-glycoprotein (the main efflux transporter of the BBB) and less active efflux of the drug from the central nervous system; while osimertinib is a moderate substrate of P-glycoprotein, intracranial drug distribution is therefore limited. Notably, there were great individual differences between 0.34% and 30.86% for almonertinib and between 1.21% and 6.29% for osimertinib. Similar wide inter-individual variability in CSF penetration has been noted in previous reports (Irie et al., 2019; Leeuw et al., 2023), suggesting that this phenomenon is not drug-specific but rather reflects patient-specific factors such as blood-brain barrier integrity, efflux transporter activity, and disease burden. This large individual variability cannot be fully reflected by plasma drug concentrations alone, suggesting that plasma drug concentrations are insufficient to predict intracranial drug exposure in patients with brain metastases.

### 4.3 Distribution and efficacy correlation

Analysis of seven patients confirmed that osimertinib achieved therapeutic plasma concentrations and penetrated the CNS (Leeuw et al., 2023; Xu et al., 2024; Li et al., 2023; Li et al., 2022a). Significant inter-individual variability in exposure was noted, potentially linked to dosing, comorbidities, or pharmacokinetic factors (Ying et al., 2020; Zheng et al., 2018). Cerebrospinal fluid concentrations (1.54–24.88 ng/mL) and penetration ratios (1.21%–6.29%) demonstrated effective BBB crossing (Shim et al., 2024; Gulikers et al., 2023). Imaging correlation showed that patients with higher CSF exposure (O2, O3) achieved partial response (PR) in brain metastases, while those with lower exposure (O4, O7) showed stable disease (SD). Interestingly, patients O5 and O6, despite having low plasma concentrations (67.9 and 45.3 ng/mL, respectively), achieved PR in lung lesions and SD in brain, suggesting potential differential sensitivity between systemic and intracranial disease or variability in individual drug disposition. The observed imaging improvements, tumor shrinkage or stability in lung and brain lesions, correlated with measurable cerebrospinal fluid drug concentrations (1.54–24.88 ng/mL) and penetration ratios (1.21%–6.29%). This reinforced that osimertinib’s clinical efficacy in brain metastases was underpinned by its ability to achieve therapeutic CNS exposure. Overall, the data support the role of CNS penetration in intracranial efficacy (Tozuka et al., 2023; Zhang et al., 2023b; Zhao et al., 2021).

Almonertinib exhibited considerable inter-patient variability in plasma and cerebrospinal fluid concentrations. Higher cerebrospinal fluid levels and penetration ratios were associated with better intracranial responses (PR on MRI), while lower CNS exposure correlated with progressive disease (PD). This underscored the importance of CSF concentration monitoring for efficacy assessment (INTERNATIONAL AGENCY FOR RESEARCH ON CANCER, 2020; Schenk et al., 2021; Yang et al., 2020). Variability might arise from differences in BBB function, metabolism, or drug interactions (Nagasaka et al., 2021). Notably, patient A10, who achieved a PR in lung lesions but experienced PD in the brain, had a remarkably low CSF concentration (1.1 ng/mL) and penetration rate (0.44%), further demonstrating that adequate CNS drug exposure is critical for intracranial disease control and that plasma concentration alone is an unreliable surrogate for CNS efficacy. Among the 13 patients, brain MRI responses were distributed as PR (n = 3), SD (n = 7), and PD (n = 3); patients achieving PR had significantly higher CSF concentrations and penetration ratios compared to those with SD or PD (Figures 3E,F). Combination therapies (e.g., with anti-angiogenics) might alter BBB permeability and CNS exposure, requiring tailored strategies (Zhang et al., 2021; Xiong et al., 2017; Tang et al., 2020). Future research should focus on exposure-response modeling, combination therapy efficacy, and the impact of resistance mechanisms to guide personalized treatment for brain metastases (Aghai-Trommeschlaeger et al., 2022; Li et al., 2022b; Zhou et al., 2021; Zhang et al., 2021b).

### 4.4 Clinical application and imaging correlation

Integrating concentration data with imaging provided a rationale for optimizing treatment in advanced NSCLC (Si et al., 2024; Yu et al., 2023). The correlation analysis between drug concentration and imaging efficacy showed that there was a significant separation between systemic treatment response and intracranial treatment response. Steady-state trough plasma concentrations were not significantly associated with the degree of lung primary tumor shrinkage in either osimertinib or almonertinib, which may explain in part why some patients with adequate plasma drug exposure still experience intracranial lesion progression. For intracranial lesions, patients with brain metastases achieving partial response (PR) had significantly higher cerebrospinal fluid drug concentration and drug permeability than patients with stable disease (SD) or progressive disease (PD), and the difference was particularly prominent in the almonertinib group (P < 0.001 for cerebrospinal fluid concentration). Typical cases further validate this conclusion: partial response of intracranial lesions was achieved in O2 and O3 patients treated with osimertinib with very high cerebrospinal fluid drug penetration levels; in contrast, A10 patients treated with almonertinib had satisfactory response of pulmonary lesions, but intracranial lesions progressed, with cerebrospinal fluid drug concentrations of only 1.1 ng/mL and drug penetration as low as 0.44%. A13 presented with meningeal metastasis and minimal intracranial drug accumulation, further confirming that inadequate cerebrospinal fluid drug exposure is a core risk factor for intracranial treatment failure. The above findings support the conclusion that the control of intracranial tumors mainly depends on whether the drug can achieve effective distribution in the cerebrospinal fluid space, rather than peripheral plasma drug concentration. Traditional therapeutic drug monitoring (TDM) protocols that rely solely on plasma monitoring may underestimate the risk of central nervous system lesion progression in patients with brain metastases.

### 4.5 Limitations of study and future perspective

This study has limitations. First, this was a single-center retrospective analysis with a relatively small sample size (7 osimertinib-treated patients and 13 almonertinib -treated patients); larger multicenter prospective cohort studies are needed to verify the association between cerebrospinal fluid concentration and intracranial efficacy. Secondly, CSF samples were collected at a fixed steady state time point, and CSF drug concentrations were not dynamically monitored throughout the treatment, so changes in intracranial drug concentrations during long-term medication could not be reflected. Third, we did not detect the expression of drug efflux transporters (P-gp, BCRP) in the brain tissue of patients, which would not further elucidate the molecular mechanisms leading to individual differences in drug permeability. Finally, this study only uses short-term imaging response as the efficacy evaluation index; subsequent studies should include long-term survival indicators such as intracranial progression-free survival. Future research interests include: (1) recruiting a large prospective cohort to identify clinical cerebrospinal fluid concentration thresholds associated with sustained control of intracranial disease; (2) collecting plasma and cerebrospinal fluid samples regularly throughout treatment to understand trends in blood-brain barrier drug permeability; (3) detecting efflux transporter genotypes at the same time to identify genetic factors affecting the intracranial distribution of TKIs; (4) conducting interventional clinical trials to verify whether dose adjustment based on cerebrospinal fluid drug concentration monitoring results can improve the intracranial disease control rate in patients with EGFR mutant brain metastases; (5) herapeutic drug monitoring combined with genotyping could guide personalized dosing, especially in patients with leptomeningeal disease or rare EGFR mutations (Xiong, 2023).

## 5 Conclusion

In this study, a reliable UPLC-MS/MS analytical method was developed and validated for the simultaneous determination of osimertinib and almonertinib concentrations in human plasma and cerebrospinal fluid. All parameters of the assay met the bioanalytical criteria and therefore accurately detected clinical samples from NSCLC patients with brain metastases. The exposure-efficacy analysis showed that there was a difference between the effect of the drug *in vivo* and its effect in the brain: the drug concentration in plasma was not associated with the remission of lung lesions, while the drug concentration in cerebrospinal fluid and the ability of the drug to penetrate the blood-brain barrier were essential for the control of brain metastases. Insufficient drug concentration in cerebrospinal fluid can lead to intracranial disease progression and meningeal metastasis. In summary, drug concentration in cerebrospinal fluid is a more reliable biomarker to assess the intracranial efficacy of EGFR-TKI drugs compared to plasma concentration. Relying on plasma concentrations alone for drug monitoring does not accurately predict the prognosis of intracranial therapy. This study provides preliminary evidence to support the development of personalized medication regimens and drug concentration monitoring strategies for EGFR mutant NSCLC patients with brain metastases. However, larger prospective studies are warranted to confirm these findings and establish definitive clinical recommendations.

## 6 Non-specialist summary

This study helps doctors personalize treatment for advanced lung cancer patients with brain metastases. We developed a sensitive method to measure concentrations of two targeted drugs (osimertinib and almonertinib) in patients’ blood and cerebrospinal fluid. Results from 20 patients show that higher drug levels in the cerebrospinal fluid, not in the blood, correlate with better control of brain tumors. Monitoring drug concentration in this brain fluid could guide more effective, individualized treatment plans for these patients.

## Data Availability

The original contributions presented in the study are included in the article/supplementary material, further inquiries can be directed to the corresponding author.

## References

[B1] Aghai-Trommeschlaeger F. Zimmermann S. Gesierich A. Kalogirou C. Goebeler M. E. Jung P. (2022). Comparison of a newly developed high performance liquid chromatography method with diode array detection to a liquid chromatography tandem mass spectrometry method for the quantification of cabozantinib, dabrafenib, nilotinib and osimertinib in human serum - application to therapeutic drug monitoring. Clin. Biochemistry 105-106, 35–43. 10.1016/j.clinbiochem.2022.04.011 35483452

[B2] Guan S. X. Shi W. Zhao Z. R. Wang F. Jiang F. L. Zhang C. B. (2019). Determination of apatinib and its three active metabolites by UPLC-MS/MS in a phase IV clinical trial in NSCLC patients. Bioanalysis 11 (22), 2049–2060. 10.4155/bio-2019-0214 31829738

[B3] Gulikers J. L. Dursun S. Veerman M. G. D. Jebbink M. Kruithof P. D. Steendam C. M. J. (2023). 1347P peripheral osimertinib (osi) plasma trough concentration (Cmin,SS) as surrogate parameter for development and progression of brain metastasis (BM) in patients (pts) with EGFR+ advanced non-small cell lung cancer (aNSCLC). Ann. Oncol. 34 (S2), S775. 10.1016/j.annonc.2023.09.2380

[B4] Hsu C. P. Chang C. W. J. Chiu C. L. Yang C. T. Kuo S. C. H. Fang Y. F. (2025). Analysis of genomic alternations in epidermal growth factor receptor (EGFR)-T790M-mutated non-small cell lung cancer (NSCLC) patients with acquired resistance to osimertinib therapy. Clin. Transl. Oncol. 2024, (prepublish) 27, 1–13. 10.1007/s12094-024-03727-7 39317868 PMC12033095

[B5] INTERNATIONAL AGENCY FOR RESEARCH ON CANCER (2020). Global Cancer Observatory: Cancer Today [EB/OL], 12.

[B6] Irie K. Nanjo S. Hata A. Yamasaki Y. Okada Y. Katakami N. (2019). Development of an LC-MS/MS-based method for quantitation of osimertinib in human plasma and cerebrospinal fluid. Bioanalysis 11 (9), 847–854. 10.4155/bio-2018-0292 31084202

[B7] Kadyrbayeva R. Kaidarova D. Shatkovskaya O. Goncharova T. Orazgalieva M. Ossikbayeva S. (2024). EGFR T790M mutation detection in NSCLC patients resistant to tyrosine kinase inhibitor therapy. Panminerva Medica 66, 372–379. 10.23736/S0031-0808.24.05172-3 39328055

[B8] Leeuw S. P. D. Bruijn P. D. Koolen S. L. W. Dingemans A. M. C. Mathijssen R. H. J. Veerman G. D. M. (2023). Quantitation of osimertinib, alectinib and lorlatinib in human cerebrospinal fluid by UPLC-MS/MS. J. Pharm. Biomed. Analysis 225, 115233. 10.1016/j.jpba.2023.115233 36638566

[B9] Li Y. Meng L. Ma Y. L. Li Y. J. Xing X. Q. Guo C. H. (2022a). Determination of osimertinib, aumolertinib, and furmonertinib in human plasma for therapeutic drug monitoring by UPLC-MS/MS. Molecules 27 (14), 4474. 10.3390/molecules27144474 35889345 PMC9325192

[B10] Li Y. Zhi X. R. Xing X. Q. An J. Guo C. H. Bai W. J. (2022b). el at. Determination of almonertinib in human plasma by liquid chromatography-tandem mass spectrometry. Chin. J. Clin. Pharmacol. 38 (13), 1531–1534. 10.12092/j.0254-1716.2022.13.1531

[B11] Li B. Chen W. Q. Fang L. Wang X. X. Qin W. Li S. (2023). A fully validated method for simultaneous determination of icotinib, osimertinib, gefitinib and O-desmethyl gefitinib in human plasma using UPLC-MS/MS for therapeutic drug monitoring. J. Pharm. Biomed. Analysis 236, 115697. 10.1016/j.jpba.2023.115697 37713984

[B12] Liu Z. C. Li Z. X. Zhang Y. Zhou T. Zhang J. Y. You W. C. (2021). Interpretation on the report of global cancer statistics 2020. J. Multidiscip. Cancer Manag. (Electron. Ver.). 7 (2), 1–14. 10.12151/JMCM.2021.02-01

[B13] Ma J. Zhang P. Wang Y. N. Liu M. D. Cao K. Wei S. S. (2024). LncRNA HAR1A inhibits non-small cell lung cancer growth by downregulating c-MYC transcripts and facilitating its proteasomal degradation. Int. Immunopharmacol. 142 (PB), 113264. 10.1016/j.intimp.2024.113264 39340992

[B14] Nagasaka M. Zhu V. W. Lim S. M. Greco M. Wu F. Y. Ou S. H. I. (2021). Beyond osimertinib: the development of third-generation EGFR tyrosine kinase inhibitors for advanced EGFR+ NSCLC. J. Thorac. Oncol. 16 (5), 740–763. 10.1016/j.jtho.2020.11.028 33338652

[B15] Nanjo S. Hata A. Okuda C. Kaji R. Okada H. Tamura D. (2018). Standard-dose osimertinib for refractory leptomeningeal metastases in T790M-positive EGFR-mutant non-small cell lung cancer. Br. J. Cancer 118 (1), 32–37. 10.1038/bjc.2017.394 29190637 PMC5765232

[B16] Nishioka N. Imai H. Endo M. Notsu A. Doshita K. Igawa S. (2024). Real-world data on subsequent therapy for first-line osimertinib-induced pneumonitis: safety of EGFR-TKI rechallenge (Osi-risk study TORG-TG2101). Target. Oncology 19 (3), 423–433. 10.1007/s11523-024-01048-x 38613731 PMC11111546

[B17] Ok O. Lee M. Kim N. Cho J. Hong S. Y. Nam M. S. (2024). Efficacy on symptoms and mortality day vs. night administration of EGFR-TKIs for advanced non-small cell lung cancer. Support. Care Cancer 32 (10), 645. 10.1007/s00520-024-08839-y 39251492

[B18] Pang L. L. Huang Y. H. Zhuang W. T. Zhang Y. X. Liao J. Hao Y. (2024). Co-occurring EGFR p.E709X mutation mediates primary resistance to the third-generation EGFR-TKIs in EGFR p.G719X-mutant patients with advanced NSCLC. Clin. Cancer Research An Official Journal Am. Assoc. Cancer Res. 30, 2636–2646. 10.1158/1078-0432.CCR-23-3302 38578683

[B19] Schenk E. L. Patil T. Pacheco J. Bunn P. A. (2021). 2020 innovation-based optimism for lung cancer outcomes. Oncologist 26 (3), e454–e472. 10.1002/onco.13590 33179378 PMC7930417

[B20] Shen G. Shi L. Tian X. Huang D. P. Chen G. Gao C. (2021). Case report: response to almonertinib in a patient with metastatic NSCLC resistant to osimertinib due to acquired EGFR L718Q mutation. Front. Pharmacol. 12, 12731895. 10.3389/fphar.2021.731895 34987382 PMC8721274

[B21] Shim B. Y. Oh C. B. Lee K. E. Park H. M. (2024). Comparison of partitioned survival modeling with state transition modeling approaches with or without consideration of brain metastasis: a case study of osimertinib *versus* pemetrexed-platinum. BMC Cancer 24 (1), 189. 10.1186/s12885-024-11971-x 38336654 PMC10858528

[B22] Si X. Y. Wang H. P. Zhang X. T. Wang M. Z. Zhang L. (2024). Efficacy and safety of furmonertinib in osimertinib-resistant patients with advanced non-samll cell lung cancer: 16 cases. Chin. J. New Drugs 33 (07), 678–681. 10.3969/j.issn.1003-3734.2024.07.008

[B23] Soin S. Ibrahim R. Wig R. Mahmood N. Pham H. N. Sainbayar E. (2024). Lung cancer mortality trends and disparities: a cross-sectional analysis 1999-2020. Cancer Epidemiology 92, 92102652. 10.1016/j.canep.2024.102652 39197399 PMC11414020

[B24] Tang Y. J. Wang S. W. Sun T. Wang F. (2020). Simultaneous determination of osimertinib and gefitinib in lung cancer patients’plasma by HPLC. China Pharm. 23 (09), 1755–1760.

[B25] Tozuka T. Noro R. Miyanaga A. Nakamichi S. Takeuchi S. Matsumoto M. (2023). Osimertinib early dose reduction as a risk to brain metastasis control in EGFR-mutant non-small cell lung cancer. Cancer Medicine 12 (17), 17731–17739. 10.1002/cam4.6393 37691552 PMC10524078

[B26] Xi N. Zhao G. R. Chen M. Zhang C. Wang Y. W. Wu S. R. (2020). Concentration determination of first-line anti-tuberculosis drugs in human cerebrospinal fluid by high performance liquid chromatography-mass spectrometry/mass spectrometry. Eval. Analysis Drug-Use Hospitals China 20 (01), 69–72.

[B27] Xiong C. Q. (2023). Analysis of the Factors Influencing the Survival Difference in the Treatment of T790M Positive Lung Adenocarcinoma with Osimertinib. Master’s thesis Kunming: Kunming Medical University.

[B28] Xiong S. Deng Z. P. Sun P. L. Mu Y. L. Xue M. X. (2017). Development and validation of a rapid and sensitive LC-MS/MS method for the pharmacokinetic study of osimertinib in rats. AOAC Int. 100 (6), 1771–1775. 10.5740/jaoacint.16-0362 28534470

[B29] Xu Y. X. Qie H. X. Zhao H. P. Gao X. N. Gao J. L. Feng Z. Y. (2024). Simultaneous determination of icotinib, osimertinib, aumolertinib, and anlotinib in human plasma for therapeutic drug monitoring by UPLC-MS/MS. J. Pharmaceutical Biomedical Analysis, 251 251116445. 10.1016/j.jpba.2024.116445 39214029

[B30] Yang P. D. (2023). Establishment of UPLC-MS Method for Detecting Erlotinib/Osimertinib Concentration and Study on the Mechanism of Osimertinib Resistance. Master’s thesis. Shijiazhuang: Hebei Medical University.

[B31] Yang J. C. H. Camidge D. R. Yang C. T. Zhou J. Y. Guo R. H. Chiu C. H. (2020). Safety, efficacy, and pharmacokinetics of aumolertinib (HS-10296) in pretreated patients with EGFR-mutated advanced NSCLC: a multicenter, open-label, phase 1 trial. J. Thorac. Oncol. 15 (12), 1907–1918. 10.1016/j.jtho.2020.09.001 32916310

[B32] Ying Z. Z. Wei J. Y. Liu R. J. Zhao F. Yu Y. F. Tian X. (2020). An UPLC-MS/MS method for determination of osimertinib in rat plasma: application to investigating the effect of ginsenoside Rg3 on the pharmacokinetics of osimertinib. Int. J. Anal. Chem. 2020, 2020. 10.1155/2020/8814214 33456471 PMC7785372

[B33] Yu Z. H. Huang Z. Z. Chen Q. X. (2023). The therapeutic advantage of ohitinib in the treatment of non-small cell lung cancer with brain metastases compared with one or two representative skin growth factor receptor-tyrosine kinase inhibitors. China J. Pharm. Econ. 18 (12), 96–99. 10.12010/j.issn.1673-5846.2023.12.022

[B34] Zhang M. Quan H. Fu L. Li Y. Lou L. (2021). Third-generation EGFR inhibitor HS-10296 in combination with famitinib, a multi-targeted tyrosine kinaseinhibitor, exerts synergistic antitumor effects through enhanced inhibition of downstream signaling in EGFR-Mutant non-small cell lung cancer cells. Thorac. Cancer 12 (8), 1210–1218. 10.1111/1759-7714.13902 33656275 PMC8046080

[B35] Zhang Y. H. Zhang Y. S. Niu W. W. Ge X. M. Huang F. H. Pang J. L. (2021a). Experimental study of almonertinib crossing the blood-brain barrier in EGFR-mutant NSCLC brain metastasis and spinal cord metastasis models. Front. Pharmacol. 12, 12750031. 10.3389/fphar.2021.7500311 PMC8497791 34630120

[B36] Zhang Y. H. Zhang Y. S. Niu W. W. Ge X. M. Huang F. H. Pang J. L. (2021b). Experimental study of almonerti nib crossing the blood-brain barrier in EGFR-mutant NSCLC brain metastasis and spinal cord metastasis models. Front. Pharmacol. 12, 750031. 10.3389/fphar.2021.750031 PMC8497791 34630120

[B37] Zhang Q. C. Xie P. Hou X. M. Zhao C. P. Duan L. Qiao H. (2023a). Benefit from almonertinib after osimertinib treat EGFR 19 exon deletion NSCLC induced severe rash: a case report. J. Chemotherapy (Florence, Italy) 36 (4), 1–9. 10.1080/1120009X.2023.2276574 37908195

[B38] Zhang L. You Y. H. Liu X. L. Liu F. J. Nie K. K. Ji Y. X. (2023b). Osimertinib combined with bevacizumab as the first-line treatment in non-small cell lung cancer patients with brain metastasis harboring epidermal growth factor receptor mutations. Thorac. Cancer 14 (15), 1355–1361. 10.1111/1759-7714.14880 37016906 PMC10212661

[B39] Zhao X. Wang B. Xie K. Z. Liu J. Y. Zhang Y. Y. Wang Y. J. (2018). Development and comparison of HPLC-MS/MS and UPLC-MS/MS methods for determining eight coccidiostats in beef. J. Chromatogr. B 1087-1088, 1087. 10.1016/j.jchromb.2018.04.044 29730532

[B40] Zhao Y. Li S. Y. Yang X. Chu L. Wang S. P. Tong T. (2021). Overall survival benefit of osimertinib and clinical value of upfront cranial local therapy in untreated EGFR-mutant non-small cell lung cancer with brain metastasis. Int. Journal Cancer 150 (8), 1318–1328. 10.1002/ijc.33904 34914096

[B41] Zheng X. Wang W. C. Zhang Y. B. Ma Y. X. Zhao H. Y. Hu P. (2018). Development and validation of a UPLC-MS/MS method for quantification of osimertinib (AZD9291) and its metabolite AZ5104 in human plasma. Biomed. Chromatography BMC 32 (12), e4365. 10.1002/bmc.4365 30119142

[B42] Zhou C. Xie L. Liu W. Zhang L. L. Zhou S. F. Wang L. (2021). Absorption, metabolism, excretion, and safety of [^14^C] almonertinib in healthy Chinese subjects. Ann. Transl. Med. 9 (10), 867. 10.21037/atm-21-1606 34164501 PMC8184462

